# Development of a framework of intervention strategies for point of care quality improvement at different levels of healthcare delivery system in India: initial lessons

**DOI:** 10.1136/bmjoq-2021-001449

**Published:** 2021-08-03

**Authors:** Vikram Datta, Sushil Srivastava, Rahul Garde, Rajesh Mehta, Nigel Livesley, Kedar Sawleshwarkar, Harish Pemde, Suprabha K Patnaik, Ankur Sooden, Mahtab Singh, Susy Sarah John, Jeena Pradeep, Anupa Vig, Achala Kumar, Vivek Singh, Vandana Bhatia, Bishan Singh Garg, Dinesh Baswal

**Affiliations:** 1 Neonatology, Kalawati Saran Children's Hospital, New Delhi, Delhi, India; 2 Neonatology, Lady Hardinge Medical College, New Delhi, Delhi, India; 3 Pediatrics, University College of Medical Sciences, Delhi, Delhi, India; 4 Quality Improvement, Nationwide Quality of Care Network, New Delhi, Delhi, India; 5 Newborn, Child and Adolescent Health, World Health Organization Regional Office for South-East Asia, New Delhi, Delhi, India; 6 Independent Health Consultant, Fremont, California, USA; 7 Neonatology, Deogiri Children's Hospital, Aurangabad, Maharashtra, India; 8 Pediatrics, Lady Hardinge Medical College, New Delhi, Delhi, India; 9 Neonatology, Bharati Vidyapeeth Deemed University Medical College, Pune, Maharastra, India; 10 QI, University Research Co LLC, Bethesda, Maryland, USA; 11 Technical Advisor Health Systems, Nationwide Quality of Care Network, Indora, Himachal Pradesh, India; 12 QI, Nationwide Quality Of Care Network India, New Delhi, Delhi, India; 13 College of Nursing, Lady Hardinge Medical College, New Delhi, Delhi, India; 14 Department of Nursing, Kalawati Saran Children's Hospital, Lady Hardinge Medical College, New Delhi, Delhi, India; 15 Telemedicine, Piramal Swasthya, Noida, NCR, India; 16 Obstetrics and Gynaecology, Piramal Swasthya, New Delhi, Delhi, India; 17 UNICEF India Country Office, Delhi, India; 18 UNICEF Madhya Pradesh, Bhopal, India; 19 Mahatma Gandhi Institute of Medical Sciences, Sevagram, Maharashtra, India; 20 Maternal Health Division, Ministry of Health and Family Welfare, Government of India, New Delhi, Delhi, India

**Keywords:** implementation science, healthcare quality improvement, health services research

## Abstract

**Background:**

Inadequate quality of care has been identified as one of the most significant challenges to achieving universal health coverage in low-income and middle-income countries. To address this WHO-SEARO, the point of care quality improvement (POCQI) method has been developed. This paper describes developing a dynamic framework for the implementation of POCQI across India from 2015 to 2020.

**Methods:**

A total of 10 intervention strategies were designed as per the needs of the local health settings. These strategies were implemented across 10 states of India, using a modification of the ‘translating research in practice’ framework. Healthcare professionals and administrators were trained in POCQI using a combination of onsite and online training methods followed by coaching and mentoring support. The implementation strategy changed to a fully digital community of practice platform during the active phase of the COVID-19 pandemic. Dashboard process, outcome indicators and crude cost of implementation were collected and analysed across the implementation sites.

**Results:**

Three implementation frameworks were evolved over the study period. The combined population benefitting from these interventions was 103 million. A pool of QI teams from 131 facilities successfully undertook 165 QI projects supported by a pool of 240 mentors over the study period. A total of 21 QI resources and 6 publications in peer-reviewed journals were also developed. The average cost of implementing POCQI initiatives for a target population of one million was US$ 3219. A total of 100 online activities were conducted over 6 months by the digital community of practice. The framework has recently extended digitally across the South-East Asian region.

**Conclusion:**

The development of an implementation framework for POCQI is an essential requirement for the initiative’s successful country-wide scale. The implementation plan should be flexible to the healthcare system’s needs, target population and the implementing agency’s capacity and amenable to multiple iterative changes.

## Introduction

As the world moves from millennium development goals to sustainable development goals (SDGs),^1^ achieving SDGs require a system thinking approach.^2^ A system thinking approach is one of the weakest links in the health systems of low-income and middle-income countries (LMICs).^3^ This weakness is further compounded by the challenge of the low quality of care (QoC) in these health systems, a significant bottleneck for ensuring universal health coverage.

India contributes the most significant chunk of the global neonatal, under-five and maternal mortality.^4^ The number of qualified doctors and combined midwives, nurses and doctor’s ratio per 10 000 population are 3.3 and 6.4, respectively, compared with 23 as advocated by the WHO.^5^ Only half of all the country’s neonatal units have a fair number of trained doctors and nurses deployed.^6^ In the last 10 years, the country’s health infrastructure and resources have seen a tremendous expansion after introducing a central government-sponsored National Rural Health Mission Programme, which is now a part of the National Health Mission (NHM).^7^ However, human resource availability continues to fall short compared with the requirements of health facilities.^8^

In 2015, WHO SEARO launched the regional framework for QoC^9^ that paved the way for the development of the point of care quality improvement (POCQI) method.^10^ One recommendation of this framework was to create systems for building QI capacity at the health facility level. A team of doctors and nurses trained in the POCQI method by WHO SEARO and USAID ASSIST scaled up the capacity building for quality improvement (QI) across India solely based on voluntary participation using an informal network structure.^11^ This training coincided with developing a QI initiative for birthing areas and special newborn care units across all government health facilities in India, known as LaQshya in 2017.^12^ The informal QoC network was formalised in 2018.^11^ The network disseminated the knowledge and skill of QI across the country in a graded manner using the POCQI method-based multiple implementation interventions. This paper describes the creation of these implementation interventions across various health system levels in India and initial observations thereof.

## Methods

### Setting

The implementation exercise was carried out across all health systems (macro-level, meso-level and micro-level corresponding to national/state-level, district-level and facility-level, respectively) across 10 states in India. The implementation facilities included the primary health centres, community health centres, district hospitals, medical and nursing colleges across the public and private sectors. These implementations were carried out across India from 2015 to 2020. Details of these intervention strategies are given in table 1.

**Table 1:** Details of QI intervention strategies

QI implementation strategy (duration)	States/districts where QI projects were undertaken under this strategy	Population (in millions) that was affected by the QI intervention(s)*	Stakeholders involved in implementing this model (besides the QI network)	Pivotal human resource	Improvement observed at (MACRO/MESO/ MICRO)
**Standalone QI support** (from 2016 to 2018 active phase, 2018 until as the sustenance phase)	Across three states in India Delhi, Maharashtra, Karnataka	3.0	Three government medical colleges, two district hospitals, three private hospitals	Facility-level care providers (like doctors, nurses, paramedics, personnel from the administration, drug/general store, pharmacy, ambulance driver, etc.	Health facility level (MICRO)
**Bottle Neck Analysis followed by introducing QI**^24^ (2015–2016 active phase; 2016–2017 sustenance phase)	Across one state Meghalaya (five districts)	3.7	State Health Department (NHM), USAID-ASSIST, QI Cell in a Medical College Hospital	Facility-level care providers, district and state health department officials	Health facility level and at state level (MICRO AND MESO)
**QI with Nursing Profession** (since August 2017 to date)	Delhi (two districts)	3.0	Continued Nursing Education (CNE) cell and QI cell of a medical college hospital	Nurses deployed in health facilities/nursing colleges.	Health facility level and nursing college level (MICRO)
**QI with Medical and Nursing students**^25^ (since March 2018–until)	Seven medical colleges and one nursing college across Delhi, Karnataka, Sikkim, Gujarat	Not applicable	Six government medical colleges, one private medical college, one nursing college and the QI cell of a medical college hospital	Undergraduate students of nursing and medical colleges across. QI Mentors form the medical and nursing college teaching hospitals	Student level - with constant and in supportive and clinical areas of the health facility (MICRO)
**State Health Department** (**NHM)-led QI for nursing students** (nursing schools/colleges) (January–March)	Madhya Pradesh (two districts)	1.9	Govt. Colleges of Nursing, Respective District Hospitals, State Health Department (NHM MP), Development partners	Undergraduate students of nursing colleges in state of Madhya Pradesh, India.	Student level - with constant and in supportive and clinical areas of the health facility (MICRO)
**Hub and Spoke model for QI (rural**)(26) (July, 2018–June, 2019. Inclusive of both active and sustenance phase)	Maharashtra (one district)	2.0	Medical college hospital, district level health facilities, NHM Maharashtra (District and State officials), WHO-SEARO, QI Cell of a medical college hospital, New Delhi	Hub facility-based mentors as focal point of handholding spoke facilities to develop their QI skills	Facility-level with development of QI linkage between tertiary care centres (medical college) and secondary care (district hospital, community health centre, etc.) (MICRO and MESO)
**Hub and Spoke model for QI (urban)(**26) (July, 2018–June, 2019. Inclusive of both active and sustenance phase)	Delhi (two districts)	3.0	NHM Delhi, Medical college hospital, district level hospitals, WHO-SEARO, QI Cell of a Medical college hospital, New Delhi	--same as above--	--same as above--
**QI mentoring integration with national perinatal care initiative in district hospitals** (September 2018–August 2019)	Madhya Pradesh (nine districts)	14.5	NHM MP, UNICEF MP	Healthcare providers (doctors, nurses, etc.) from Special newborn care units	Special newborn care unit’s level (MICRO)
**QI mentoring integration with national perinatal care initiative in teaching hospitals** (July 2019–until)	12 medical colleges, across India	71.5	Maternal Health Division, MOHFW, NHSRC, State NHM Offices, WHO-SEARO, New Delhi, UNICEF (country and state offices),	Obstetricians, Paediatricians and Senior Nurses (as part of a quality-of-care network).	Facility-level (tertiary care centres that is, medical college level) (MICRO, with constant MACRO level support)
QI mentoring integration with national perinatal care initiative (July 2019–January 2020)	Uttar Pradesh (three districts)	11.5	NHM UP, UNICEF UP	District-level quality consultants	Facility-level improvement with impact at district level (MICRO)
Online Community of Practice (Digital Platform) (Ongoing since August 2020)	Online platform with participants from around the world (USA, UK, Qatar, Bangladesh and India)	Not applicable	WHO-SEARO, Ministry of Health & Family Welfare, ISQua, BMJ India, Oxford University Hospitals, NHS, University Research Company, MGIMS, Wardha, Aastarika technologies, 3M, CAHO	QI champions from all facilities associated with the network, national and state health departments, development partners, QI teams from South Asia region.	MICRO-LEVEL, MESO-LEVEL MACRO LEVEL

*Extrapolated data for 2019 from baseline data about district populations from Census 2011.

†Aspirational districts are those districts in India, that are affected by poor socio-economic indicators. These are aspirational in the context that improvement in these districts can lead to the overall improvement in human development in India.

### Study design

**Figure 1:**
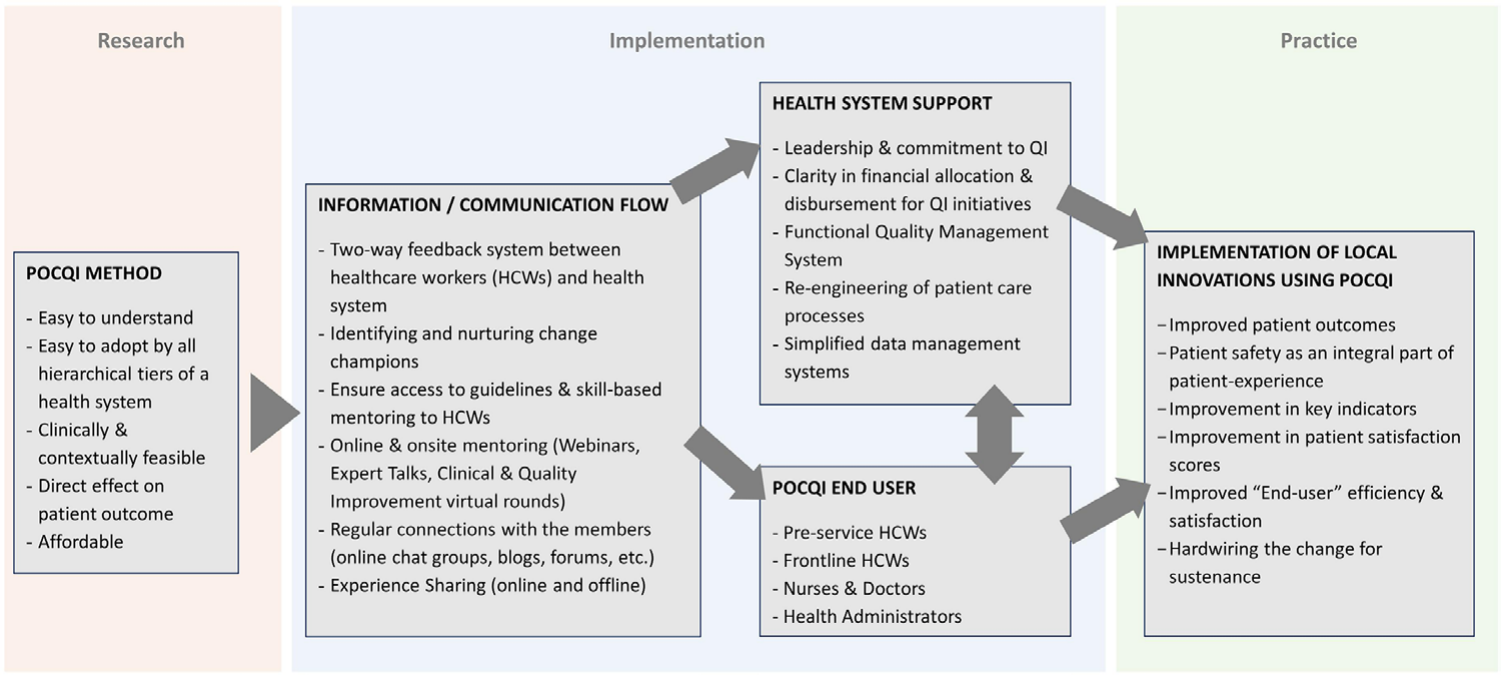
Adapted TRIP framework^13^ for implementing Point Of Care Quality Improvement (POCQI) method.

This is a descriptive observational study that used an adapted Translating Research into Practice (TRIP) framework^13^ to implement POCQI methods in a local context (figure 1).

### Implementation process

QI teams were identified using different mechanisms. The initial implementation process was driven by the voluntary participation of the local champions. The later stages were completed by nominations from the state health departments and development partners. These teams comprised healthcare professionals and workers from different health system levels and ranged from frontline community health workers to super-specialist doctors, in-service nursing professionals and nurse educators. These teams underwent training in POCQI skills and were subsequently mentored by members of the network mentioned above throughout the intervention strategies' timeframe. Mentoring was done using a mix of onsite visits and online sessions. With the onset of the COVID-19 pandemic in 2020, the online medium was widely used to continue building and mentoring the national QI teams. During the active phase of the pandemic, these online sessions led to the development of an innovative digital POCQI community of practice over the latter half of 2020.^14^

### Participants

Various healthcare workers, medical and nursing students, community members and various governmental, nongovernmental, national and international development partners were involved in implementing these strategies.

### Approvals

Approvals were obtained taken from the respective supervising authorities, which included a facility in charges, state district officials, state NHMs and ministries of health. QI team members and mentors volunteered to take part in this exercise.

### Ethical approvals

All of the changes tested in various QI intervention strategies described in this paper were about improving the implementation of widely accepted and evidence-based clinical practices. As no patient was being denied benefits from any evidence-based clinical practices, institutional review board approvals were not required.

### Data collection

The implementation exercise generated data from different levels of the health system. The implementing team’s primary data at the microlevel (facility) was collected using facility source documents, direct observations and patient interviews. Documentation of process and outcome indicators were predecided by the QI team in active consultation with the onsite mentor and central coordinator for the implementation process. The data were collected in Microsoft Excel 2016 sheets specially designed for easy use by the facility team. The data thus collected were cleaned through random cross-checks performed by the QI team leader and the QI mentor. The central coordinating team collected the data related to different intervention strategies at higher health systems (meso and macro) at the network level. This data were collated in active consultation with facility team leads and cleaned using inputs from stakeholders and development partners.

### Analyses

We analysed the data related to the key stakeholders involved, number of facilities involved, QI projects undertaken, mentors, capacity building workshops, publications and QI resources developed during the implementation process. Additionally, the average cost incurred was calculated and mapped to the population affected by the exercise’s implementation. These indicators were used to analyse the implementation process (table 2).

**Table 2:** Overview of QI capacity building done over 2015–2020

Serial number	Year	Population that would benefit from the QI initiatives* (millions)	Key stakeholders involved	Facilities involved	Number of QI projects undertaken	Number of QI workshops/ activities	Published work	QI resources developed (guidelines, case studies, etc.)	Cost per million population for implementing QI initiatives† (US$)
1	2015	1.85	2	5	0	0	0	0	3712
2	2016	1.85	3	3	5	2	0	1	–
3	2017	4.85	4	21	33	17	1	1	4808
4	2018	9.75	9	22	24	38	3	8	6521
5	2019	41.5	12	39	20	35	2	7	1630
6	2020	43	6	41	83	100‡	0	4	2644

*Approximately population of the district/region affected by the QI initiatives for maternal and newborn care.

†Includes direct costs of implementing QI initiatives by the network resources. Indirect costs of coordination, planning, developing content for QI activities, visits by partner agencies/stakeholders, etc. are not considered here.

‡Includes both onsite QI workshop and online QI and clinical mentoring sessions done for implementation of various QI initiatives.

QI, quality improvement.

### Patient involvement

No patients were involved in this work as the study’s focus was to develop intervention strategies for the POCQI initiative. Similarly, no patients were involved in developing the research questions, outcome measures, recruitment and study conduct. The results were disseminated through experience-sharing workshops to facility teams of healthcare workers, providers, funding partners and governmental agencies.

## Results

The network developed context-specific intervention strategies throughout its QI implementation experience. As a result, 10 intervention strategies for scale-up and spread of QI were implemented over 2015–2020. Details of these intervention strategies are available in the online supplemental file.

10.1136/bmjoq-2021-001449.supp1 Supplementary data

### Key features of the intervention strategies

These models were developed to overcome context-specific challenges based on differences in health settings. Context variations were about–types of learners (like healthcare students and in-service healthcare professionals), linkages between facility (standalone facility-based QI team(s) and community facility-based QI team(s) linked to teaching facility QI mentors), the geographical proximity of facilities to each other, involvement of other stakeholders like government health departments, development partners and the type of mentoring mode used—onsite, online-only or mixed mode. The disruption caused by the COVID-19 pandemic led to the development of the community of practicefor spreading QI and fostering learning among healthcare workers. The intervention strategies adopted over 2015–2020 involved multiple stakeholders and facilities and created a pool of learnings that could potentially impact nearly 103 million population (approximately 7.3% of India’s population), as cited in table 2.

These intervention strategies were stratified into four broad categories regarding different aspects of the QI initiatives:

- The tier of the health system where QI implementation occurred—government community health facilities (both primary and secondary care services), government teaching health facilities, standalone private sector health facilities. (Standalone QI support, introducing QI after QA assessment, Hub and Spoke model (rural and urban), QI mentoring integration with national perinatal care initiative in community and teaching hospitals across the country).
- The tier of human healthcare resources implementing QI initiatives—in service healthcare workers (frontline workers, nurses, doctors, administrators and other health facility staff). (Introducing QI after Bottle Neck Analysis assessment, nurse-focused QI training, medical and nursing students’ QI training, state-led nursing student’s QI training).
- Mode of engagement with QI practitioners—onsite face-to-face interactions, online interactions (to complement face-to-face interactions) or online only interactions (during the pandemic times since April 2020). (Digital community of practice (online), a general framework of other QI implementation models).
- Modes of funding—whether funded by development partners, governmental agencies, crowdfunding or voluntary self-generated funds.

### Evolution of framework

**Figure 2:**
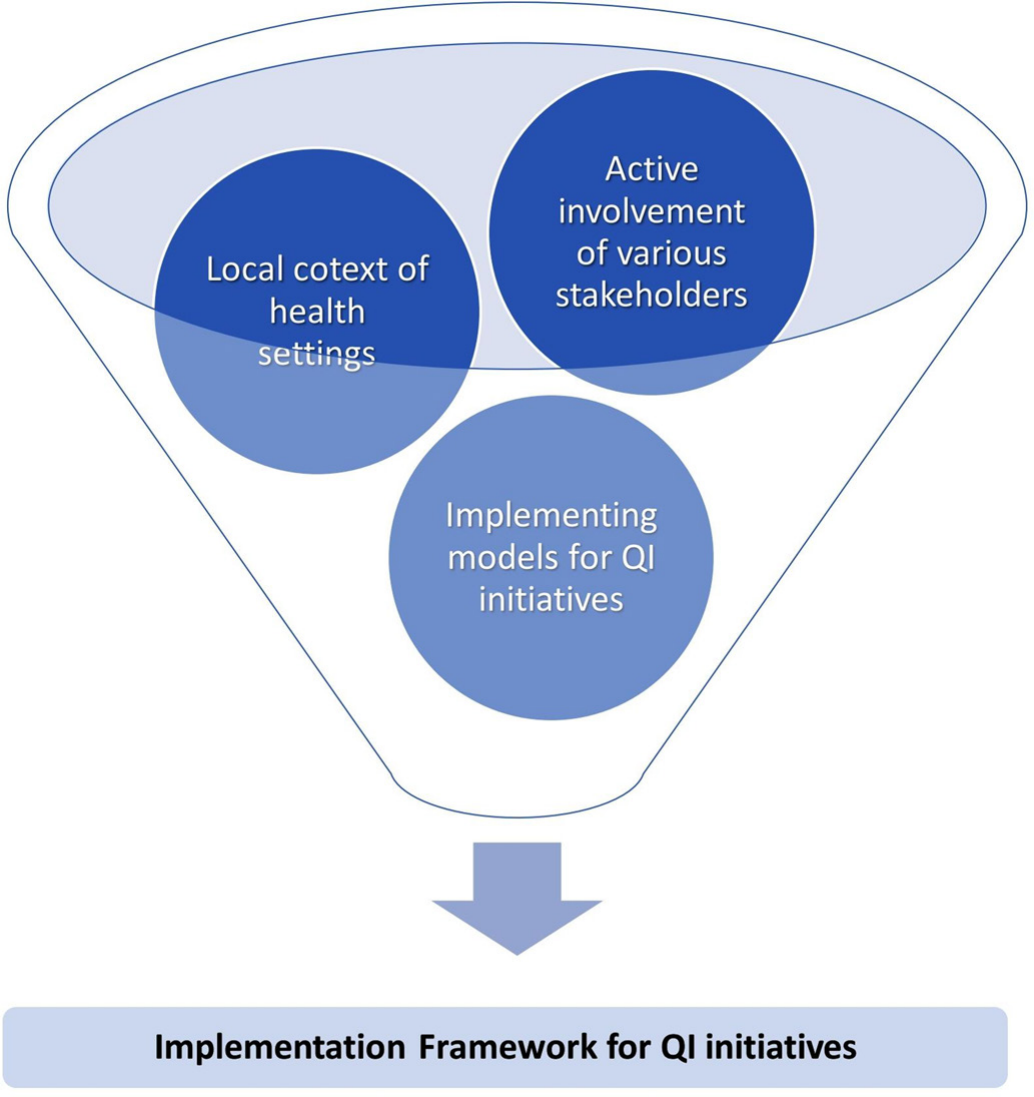
Factors influencing the development of implementation framework for QI initiatives. QI, quality improvement.

We implemented POCQI using various strategies to develop a rapidly developing framework for QI initiatives over 5 years (2015–2020). Various contextual factors influenced the QI programme implementation. The factors were (a) health facilities implementing QI, (b) stakeholders and their linkages and (c) level of the health system, that is, the microlevels, mesolevels and macrolevels (see figure 2).

**Figure 3:**
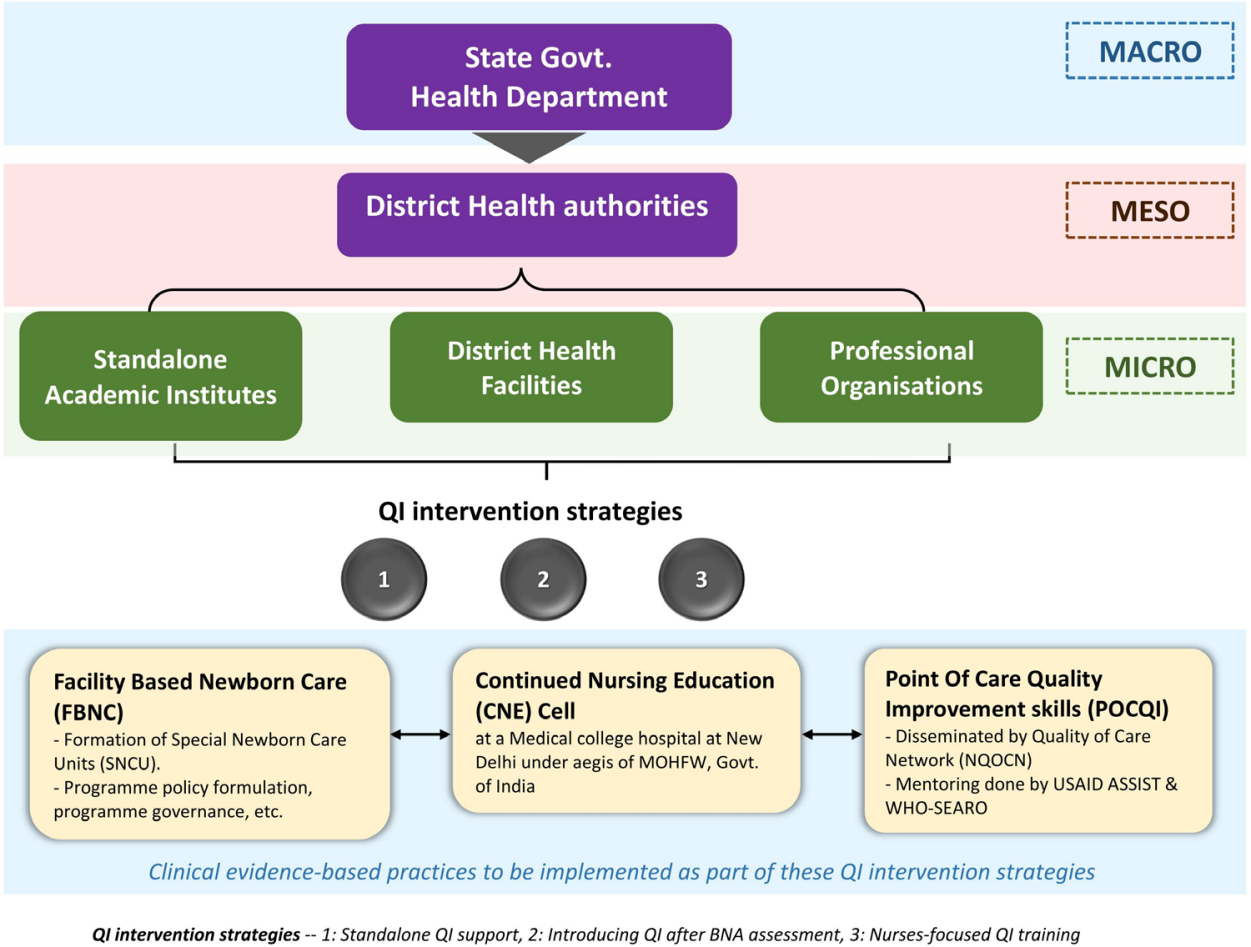
Evolution of Implementation framework—2015–17.

**Figure 4:**
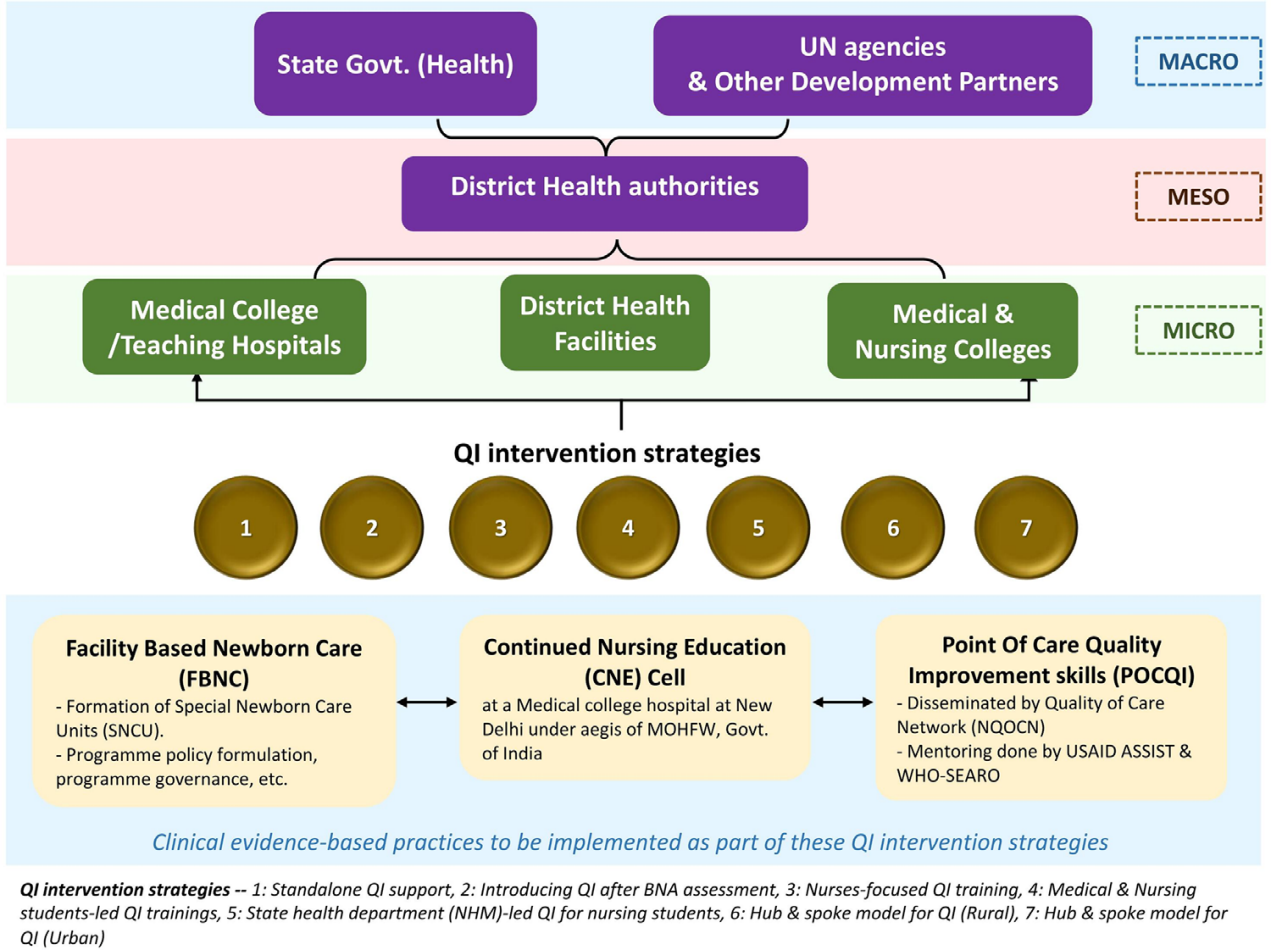
Evolution of Implementation framework—2017–18.

**Figure 5:**
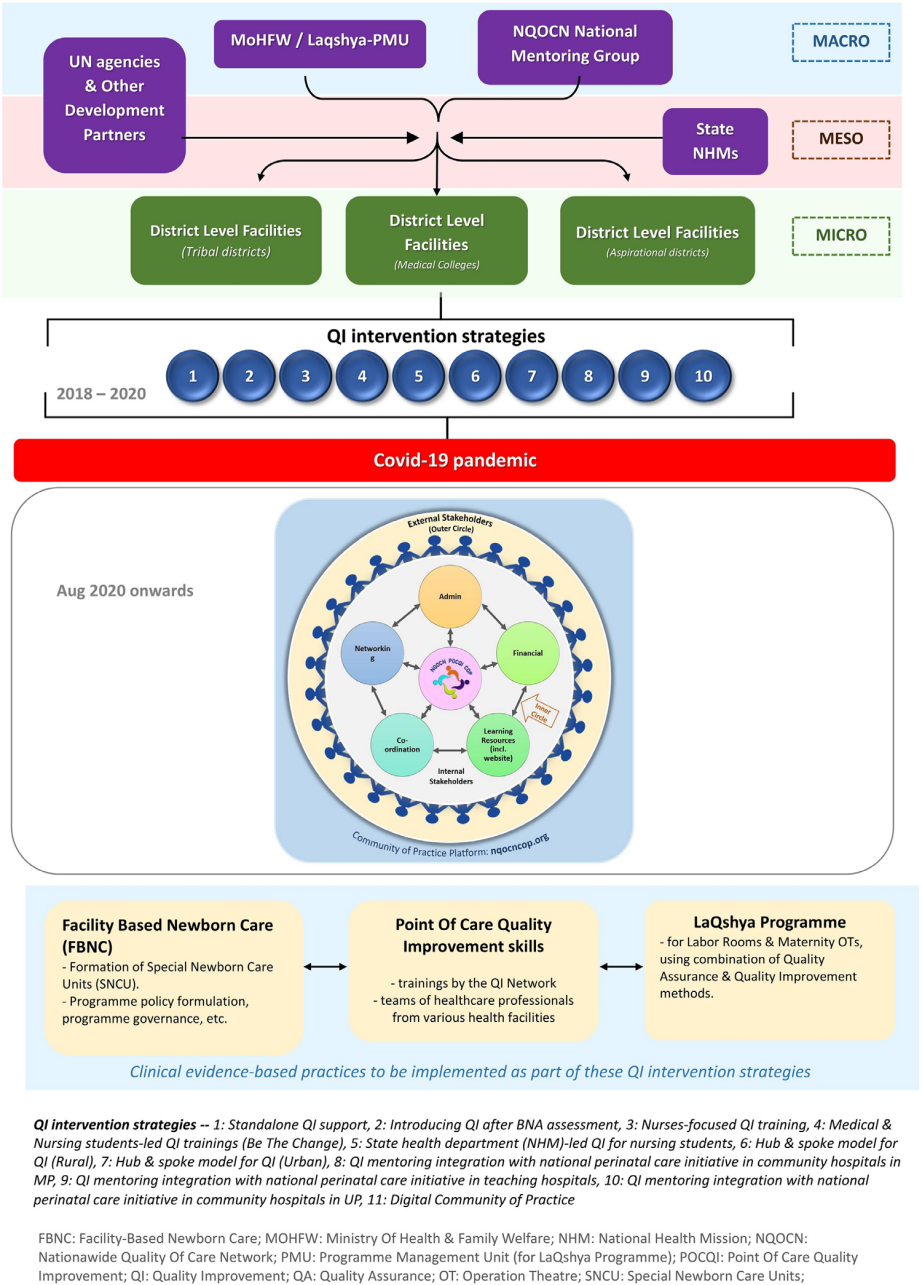
Evolution of Implementation framework—2018–20.

Various intervention strategies involving stakeholders across all health systems were used to introduce and sustain POCQI over 2015–2020, as shown in figures 3–5). The implementation framework developed rapidly in scale and scope over 2018–20 (figures 4 and 5) with the COVID-19 pandemic, the implementation strategy metamorphosed to a fully digital avatar (digital community of practice). The figures mentioned above clearly depict that POCQI implementation in an LMIC setting is a dynamic process undergoing a rapid evolution depending on the availability of resources, demands of the health system, needs of the target population in sync with the national and state health goals.

## Discussion

In 2001, the Institute of Medicine released the report ‘rossing the quality chasm’ that called for a redesign of health systems and defined the various quality elements in a healthcare setting.^15^ This need for system redesign focused on the significance of QI in bringing these changes.^16^ However, implementing QI at scale has proved to be a challenge.^17^ Numerous studies have shown that QI scale-up needs long-term leadership commitment, extensive training and support, full data recording and analysis, better human resource practices and dynamism in organisational culture for accepting new ideas.^18–20^ The factors mentioned above are a significant challenge across all healthcare delivery systems, especially in LMICs.

Partner organisations developed a simplified approach of POCQI under the leadership of WHO-SEARO to offset these challenges. The current paper describes India’s innovative intervention strategies to scale up this simplified QI approach—POCQI, across the health system’s various levels, from primary care centres to teaching hospitals.

It is well known that QI implementation and sustenance is inherently problematic because of multiple factors that can affect them.^21^ There is a need to develop intervention strategies across various levels of the health system. These interventions will potentially address various factors that can affect the successful uptake of QI initiatives.^22^ The strategies described in this paper and the ensuing frameworks developed over 5 years (2015–2020) were through a multistage, inductive process.

As shown in figures 3–5, multiple intervention strategies were field tested across India. The lessons from this exercise led us to realise the importance of interplaying multiple factors in a health system while implementing QI initiatives. These factors are of vital significance for the successful initiation and sustenance of POCQI initiatives. The authors wish to draw attention to the fact that any attempt to develop an implementation framework for QI initiatives should be open to frequent adaptations depending on the local health settings' ever-evolving needs.

This paper shows that a single strategy may not suffice to disseminate and implement QI across health systems, especially for countries with heterogeneous health systems. Therefore, implementers should have a flexible approach to intervention strategies for QI initiatives. At times, the planned strategies might need to be modified or entirely abandoned for a new one to roll out a QI initiative.

A recent scoping review of quality management models similarly highlighted a lack of implementation models for undertaking QI initiatives.^23^ The initial lessons from this implementation exercise will significantly contribute to this nascent knowledge and help implementation agencies and researchers accelerate QI implementation.

Challenges in the roll out of QI implementing strategies:

The suggested intervention strategies highlight the need for awareness about the context of specific factors for administrators and QI teams. It guides appropriate actions that can lead to positive outcomes in a health setting. Key challenges are described below:

1. Incomplete documentation and lack of robust data-keeping mechanisms cost significant time and energy at the level of the implementing team.
2. Supply chain issues and the nonavailability of essential equipment and resources were significant challenges encountered by the implementing teams.
3. Hierarchical or organisational barriers, lack of inter and intradepartmental communication can hamper synergistic QI efforts across interlinked clinical domains.
4. Frequent transfers of doctors, nurses and other HCWs involved in the QI process often derailed the improvement team’s efforts.
5. Inadequate capacity building of nurses in health facilities due to the absence of a dedicated continued nursing education programme hampered the QI initiative.
6. Lack of awareness of health facility staff regarding national and state programme guidelines and their implementation plan led to piecemeal implementation. This lack of awareness often led to confusion among facility health staff and adversely impacted the QoC provided to patients in these settings.
7. Sustenance of the QI project beyond the project duration was challenging due to a lack of resources and accountability.

### Limitations

A limitation of the proposed framework is a subjective description of the implementation of QI. It is a post hoc analysis of various QI initiatives across India. Thus, a formal, detailed description of individual QI projects leading to strategies generation and framework synthesis may be lacking in this narrative. However, the same has been reported in the published literature by the network.^11 24–26^ A formal impact assessment has not been carried out for the exercise described in this paper; however, the implementation process has been actively monitored concerning metrics mentioned before. Community participation was deficient in our intervention strategies, partly attributable to a lack of awareness and demand for high-quality care in the community. The intervention strategies evolved as a set of successful implementation initiatives undertaken across diverse clinical sociodemographic settings stacked together and spontaneously evolved into an implementation framework for the health system levels. Critics could view this spontaneous evolution as an exercise lacking planning and evaluation. However, it could be viewed as a blessing in disguise for the implementing team, as it gave them the freedom and flexibility to adapt, adopt or abandon in action. The implementation strategies were planned as per the project’s intended objectives; however, during implementation, changes were made based on the situational analysis and challenges encountered. This resulted in a modified implementation strategy. Due to the lack of uniform implementation strategy across different models, the results are not comparable.

This implementation exercise has generated valuable learnings and identified key challenges and limitations, which can be used by implementing teams in similar LMIC settings to build up implementation models of QI in challenging health settings. A recent meta-analysis concluded that models and frameworks could provide public health administrators with a choice of practical information that may be used to support capacity building efforts.^27^ Similarly, the benefits that accrue from the impact of QI initiatives for the larger population make them an essential tool for health administrators to ensure cost-effective healthcare for the community, as reported by a recent systematic review.^28^

What this study adds to the QI implementation paradigm

- The development of intervention strategies requires a mix of intuitive abilities, a clear understanding of local health systems dynamics, strong networking capacity, good communication skills, desire and a compassionate outlook towards patient care.
- Even in challenging LMIC settings, even without a framework and an implementation plan—QI work can be started using simple tools like POCQI.
- The implementation plan should be flexible to the needs of the healthcare system, target population and implementing agency/network capacity. It should be amenable to multiple iterative changes to make it appropriate for the local health settings' needs.
- The expenditure per million population to implement POCQI at scale in an LMIC like India is far more economical than many simple surgical procedures. The cost of implementing POCQI at scale for a target population of 1 million is approximately equivalent to the cost of a coronary bypass procedure in a private sector hospital in India.^29^
- As shown in this study, the process is labour intensive and requires sustained commitment to achieving the desired shift in the quality of delivered healthcare.

## Conclusion

Frameworks and models help describe and understand how interventions can be scaled up^30^ from small, individual health facility-based projects to a broader set of guidelines for a health system. Effective scaling up of such initiatives requires the systematic use of evidence and data from on-ground implementation to drive the policy and decision-making process throughout the health system–from the national level down to the community health worker level. This paper emphasises that there is no one panacea for successfully implementing QI. Each time, the implementor has to base their choice on picking the model based on local factors^31^—preparation of the site, availability of funding, development partner or government support, availability of long-term mentoring support, geographical area of implementation and social determinants of health. This fine art of balancing macrolevel, mesolevel and microlevel contexts in a setting can significantly affect seeding and spreading QI initiatives and help in a seamless implementation.

## Data Availability

Data are available upon reasonable request. All data relevant to the study are included in the article or uploaded as supplementary information. All relevant data is available upon reasonable request from the corresponding author and also available as online supplementary material.
