# Supplementary material for: Development of a framework of intervention strategies for point of care quality improvement at different levels of healthcare delivery system in India: initial lessons
Source: BMJ Open Qual. 2021 Aug 3;10(Suppl 1):e001449. doi: 10.1136/bmjoq-2021-001449 (PMC8336183; doi:10.1136/bmjoq-2021-001449)
Supplement: Supplementary data [file bmjoq-2021-001449supp001.pdf]

SUPPLEMENT

POINT OF CARE QUALITY IMPROVEMENT (POCQI) IMPLEMENTATION STRATEGIES

[1] Standalone QI support (From 2016 to 2018 active phase, 2018- till date as the sustenance phase)

This strategy derives its basis from the Quality-of-Care framework for MNH proposed by WHO. POCQI methodology is a simple 4-step method of improving processes of patient care in a context specific manner by the local teams from health facilities. This strategy brought various cadre of healthcare providers together as a team to solve their contextual problems using POCQI methodology within their existing resources. Subsequently, the QI teams were mentored to help achieve their identified aims. This strategy led to three key developments and subsequent evolution into different strategies of QI implementation. First, it is during development of this strategy, it became clear that uptake of QI skills/projects can be catalyzed with involving community also in the QI teams. This was the key learning highlighting that community is not a passive recipient of healthcare, and can be a key stakeholder for improving quality of care in health systems. Second, this strategy emphasised on training and periodic retraining's of the various functionaries in the healthcare systems in POCQI implementation skills. This empowered them to identify and address the structures, processes and outcome areas which require strengthening. Third, these empowered individuals led to creation of a pool of national coaches which effectively broadened and strengthened the spread of QI skills in various states across India.

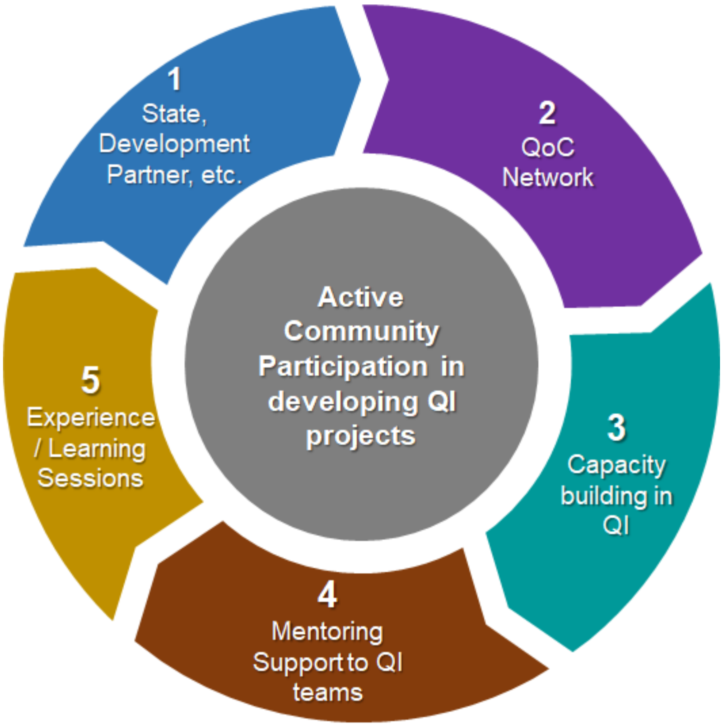

Figure a: Primary Strategy of Quality Improvement in Health Facilities.

Datta V, *et al. BMJ Open Qual* 2021; 10:e001449. doi: 10.1136/bmjoq-2021-001449

[2] Bottle Neck Analysis followed by introducing QI (2015-2016 active phase; 2016-2017 sustenance phase)

This combination strategy of using both quality assurance (QA) and quality improvement (QI) in tandem with each other was implemented in the Indian state of Meghalaya. A bottleneck analysis (BNA) was conducted in select facilities to identify the critical health system challenges. This was the first phase of the project known as the "Assessment phase". Second phase of the project was the "Improvement phase" which was implemented using POCQI methodology. In this phase, feasibility of implementing QI tools in government health facilities was tested by helping the staff develop solutions to the identified bottlenecks. Learnings from both the phases were documented and shared with the state. The project was operational from Sep 2015 to July 2017.

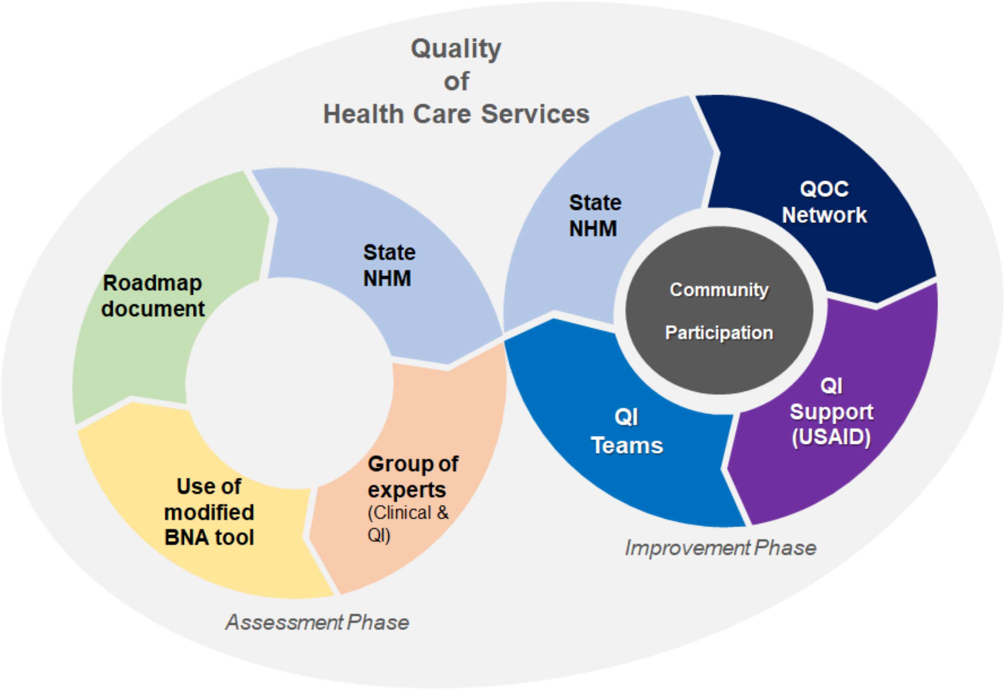

Figure b: Combined Quality Assurance and Improvement Strategy

[3] QI with Nursing Profession (Since Aug 2017 – till date)

Nurses form one of the largest pools of human resources for health. Getting them on board in any QI venture is of critical importance for its success. In order to involve more participation from nursing community (and other healthcare workers) of a health facility, network used elements of IHI's Psychology of Change framework to help the teams move beyond tokenism to an active participatory approach towards patient care. During 2017-18 network created a pool of 70 senior nurse educators, who were trained in skills of QI to handhold national teams of pre-service and in-service nurses through nursing colleges

Datta V, *et al. BMJ Open Qual* 2021; 10:e001449. doi: 10.1136/bmjoq-2021-001449

Continuing Nursing Education (CNE) programmes which were supported by MOHFW Govt. of India. As of February 2020, these nurses have helped in training and creating more than 500 strong pool of QI trained nursing professionals from across the country. These nurses, after conducting QI projects in their respective hospitals, were further trained as QI coaches and went on to support various QI teams across the country. This group of nurse QI mentors, are an important resource pool for the mentoring health facility staff for ongoing National Health Programmes, like LaQshya initiative.

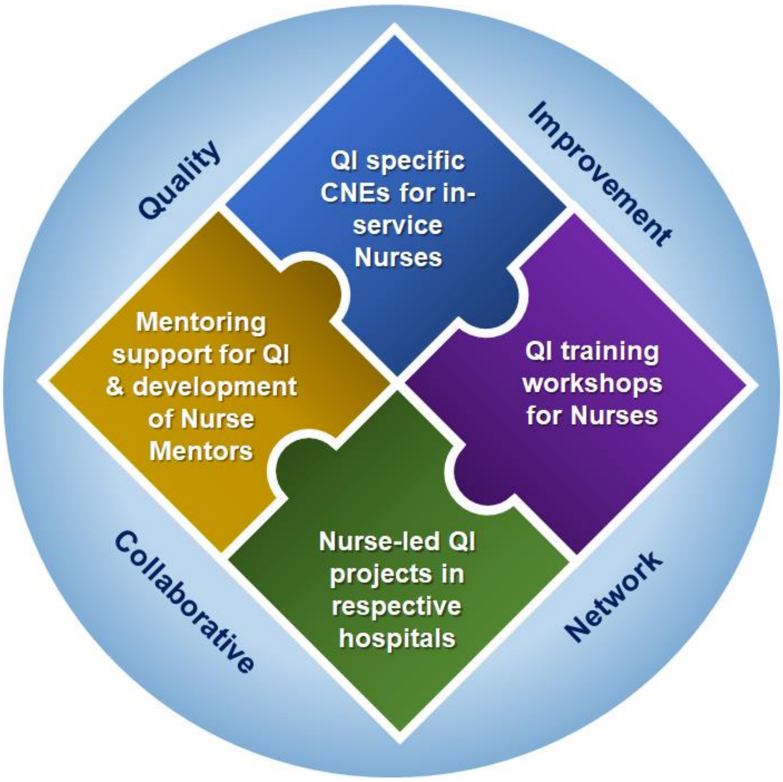

Figure c: In-Service Nurse Mentor & Continuing Nursing Education Strategy

[4] QI with Medical and Nursing students (Since Mar 2018 – till date) and

[5] State Health Department (NHM)-led QI for nursing students (Nursing Schools/ Colleges) (Jan – Mar 2019)

Students of various health care branches are an important asset for any health system. They offer the most fertile ground for institutionalizing quality consciousness into everyday life of future health care providers. Inculcating thinking about implementation of quality right from pre-service stages is a way of tackling resistance to change and innovations, which is common in health system. The network focussed on developing QI capabilities of the future generation of healthcare providers by actively engaging with undergraduate nursing and medical students. These efforts led to formation of "*Be The Change*" group, a collective of like-minded undergraduate medical and nursing students of several medical college

hospitals in India, who were trained in applying QI methodology-based problem-solving skills. Building and empowering the future generation of quality practitioners is seen by the network as one of key interventions for sustenance of future quality implementation projects. This group has been referred to in international platforms, conferences, etc. and their story is available on WHO's Quality of Care Network platform, as is a podcast highlighting their journey on the QED Networks' "Quality Talks". Learning from the success of this group, NHM MP supported the network in successfully conducting pre-service trainings at two Government Colleges of Nursing in the state of MP in 2018-2019.

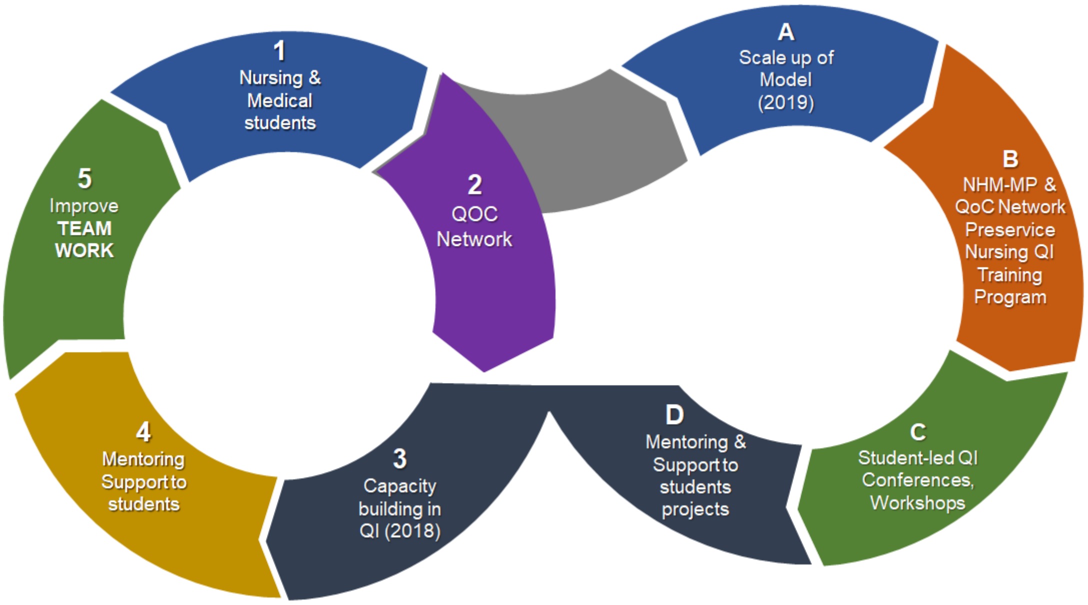

Figure d: Imparting QI skills to undergraduate medical and nursing students

[6 & 7] Hub and Spoke model for QI (rural & urban) (Jul, 2018 – Jun, 2019. Inclusive of both active and sustenance phase)

Historically, the hub and spoke strategy (HSM) has been used across various industries to augment peripheral services by centralizing key resources. This strategy was used for augmenting peripheral health facilities by connecting them to a resource replete hub. This HSM pegged a tertiary care medical college as the hub facility and the other district level or block level health facilities as the spokes. This approach had the dual benefit of developing QI practitioners (at both hub & spokes) as well as QI coaches (from hub facilities). Various elements of the HSM were: development of QI plan for the districts, QI training of facility teams, providing QI coaching support (to both hub & spokes), encouraging peer-to-peer learning and experience sharing of QI projects. In addition, efforts were made to ensure that facilities participating in QI projects were provided with an enabling environment. Most important of these included district leadership support and support system for the project

(i.e. operational funds &HR). Six of the seven facilities moved in the direction of improvement in terms of their QI aims. The overall complexity of these aims depended largely on the enabling environment available at that facility to test out various change ideas (and subsequently learn from them). This strategy represents a prototype to bridge the challenges in resource building (knowledge and capacity) even when complete decentralization is not possible. This strategy identifies the various stakeholders that help in implementing quality improvement initiatives across the macro, meso and micro levels of the health system hierarchy, where macro-level represents the national or sub-national level (e.g. a state), the meso-level is at the level of district and the micro-level is at facility or community level.

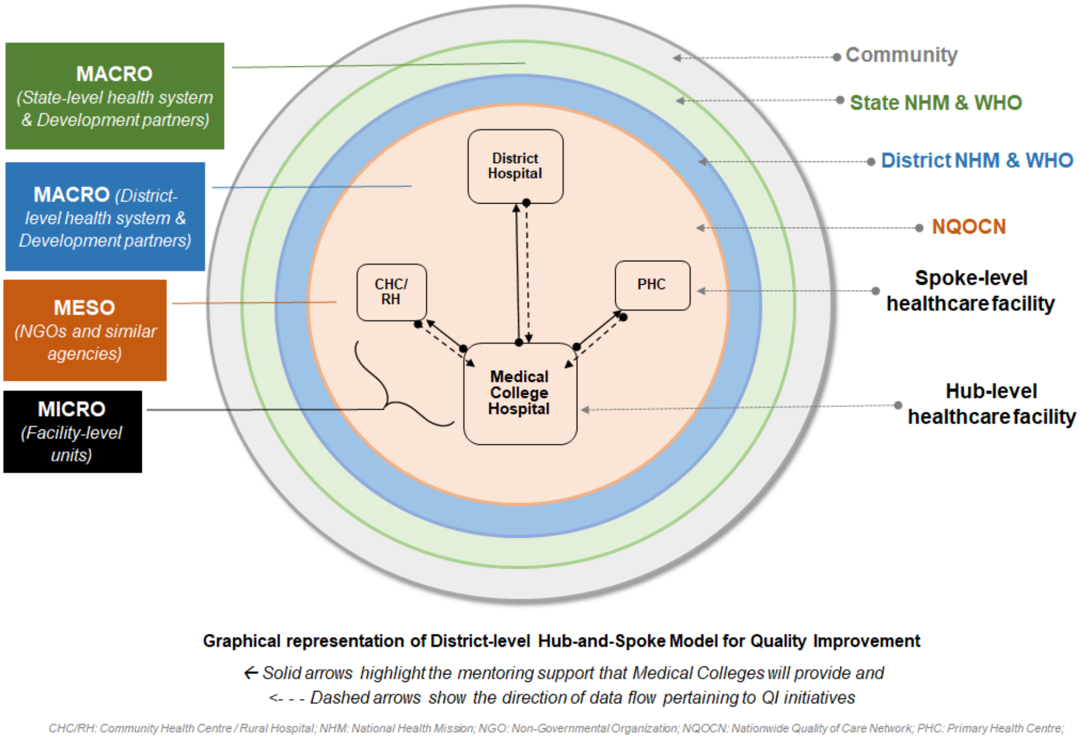

Figure e-1: Hub and Spoke Strategy for QI (plan)

Datta V, *et al. BMJ Open Qual* 2021; 10:e001449. doi: 10.1136/bmjoq-2021-001449

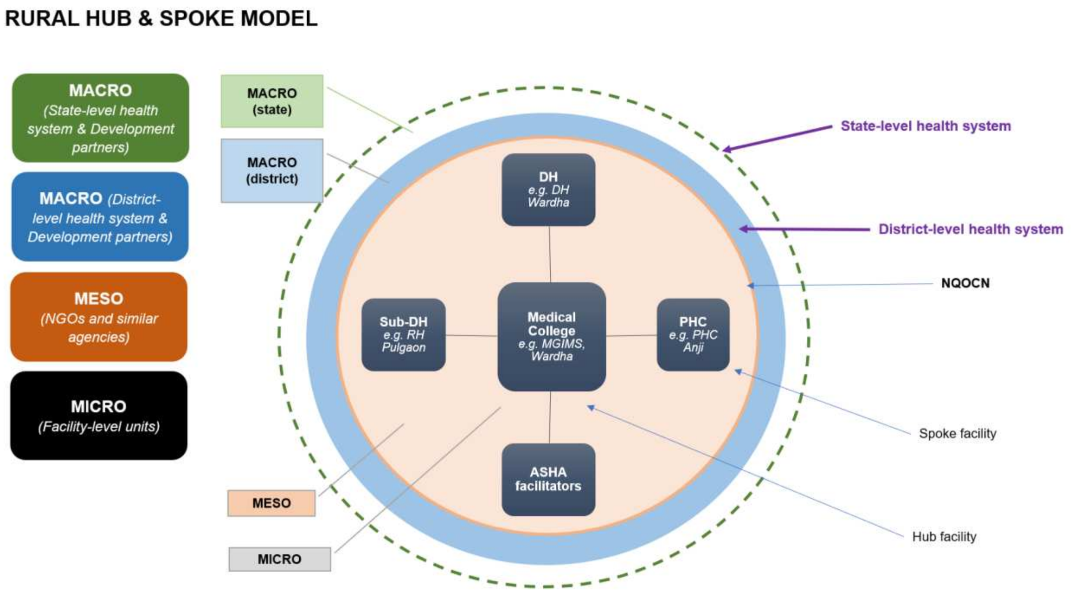

Figure e-2: Hub and Spoke Strategy for QI (rural)

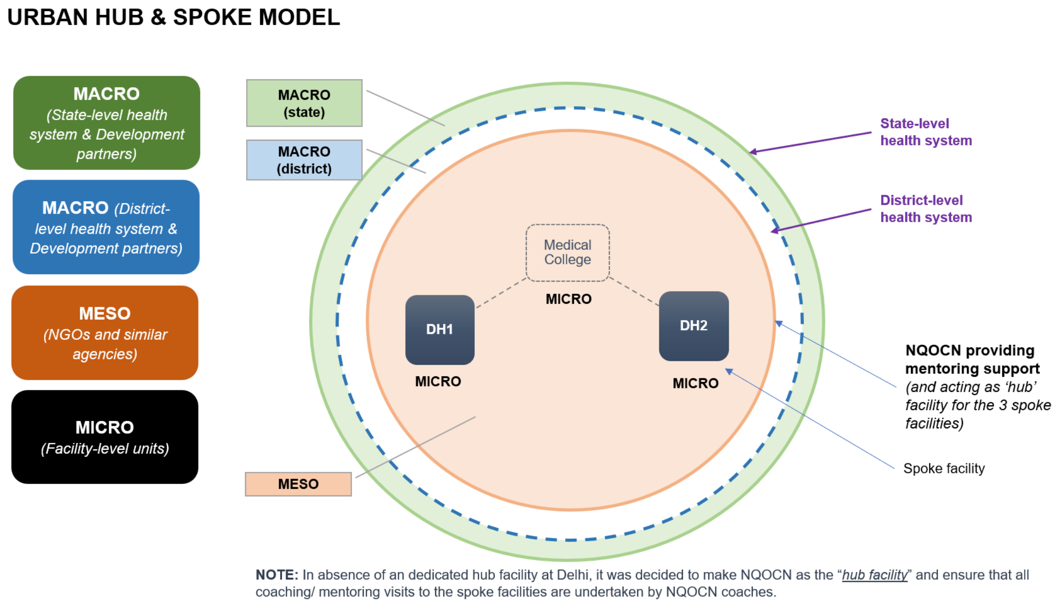

Figure e-3: Hub and Spoke Strategy for QI (urban)

[8 & 9] QI mentoring integration with national perinatal care initiative in district hospitals in state of Madhya Pradesh (Sep 2018 – Aug 2019) & Uttar Pradesh (Jul 2019 – Jan 2020)

As opposed to the Meghalaya strategy which had two phases of assessment and improvement to overcome identified system level bottlenecks, this strategy implemented in states of Uttar Pradesh (UP) and Madhya Pradesh (MP) (see Figure 7) had the network supporting the state NHMs and development partners to develop their capacity to drive QI in their health systems utilising their own internal resources. Mentors from the network, trained the health care providers from district level health facilities in both of these states. In UP, an additional set of staff was also trained in QI – i.e. the district quality consultants. Primary differentiator due to which these strategies evolved as distinct from one another was the presence of district level Quality consultants in UP strategy. These consultants would drive the required changes at the district and sub-district facilities and ensure better coordination between the state and district level functionaries in the state of UP. Coordination of this type is an important element for creating fertile conditions for uptake and sustenance of QI efforts. Besides training the facility and/or district staff in QI methodology, the network helped these respective states in creating a pool of QI mentors. In the state of UP, the district quality consultants from 35 district were trained as QI coaches to handhold the facilities in their district. In the state of MP, network collaborated with various development partners working in the state to get their district level consultants trained as QI coaches – a pool of 34 such QI coaches were trained in the state. For each facility a team of two national mentors was assigned. They were actively assisted by the state resource for QI (as mentioned above) in conducting the onsite mentoring and remote handholding of the select aspirational and tribal districts across UP & MP.

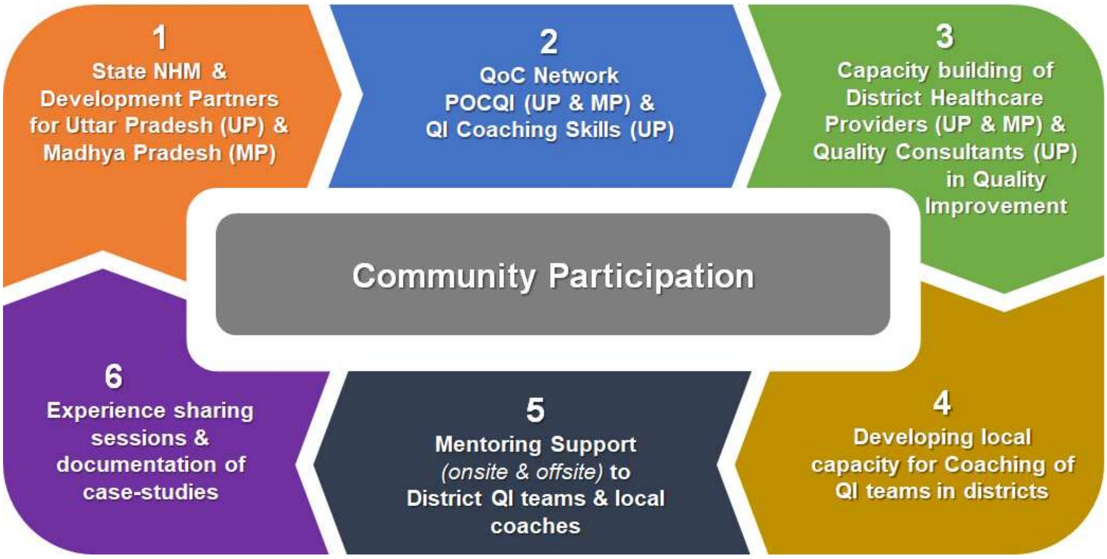

Figure f: State Specific Strategy for QI Implementation – Uttar Pradesh (UP) & Madhya Pradesh (MP)

[10] QI mentoring integration with national perinatal care initiative in teaching hospitals (Jul 2019 – till date)

LaQshya programme was launched by MOHFW, Govt. of India in 2017. The main objective of this programme was to help improve the quality of maternal care in the public health facilities across India. LaQshya wanted to achieve this aim by combining quality assurance and quality improvement methods. NOQCN was notified as technical partner to LaQshya programme to help govt. roll out the QI elements of this programme viz. Rapid Improvement Cycles (RICs). For this purpose, a national mentoring group was formed under guidance of NQOCN. Due to combining of both QA and QI in this programme, the facilities required a combination of clinical and QI mentoring during each visit to be able to help them achieve LaQshya aims. An obstetrician and a nurse mentor trained in POCQI methodology was made part of mentoring visit under NMG besides a Pediatric Mentor to fulfill this specific need of the facilities. This clinical plus QI is a new addition to the overall mentoring methods used in earlier strategies. Like other strategies, intensive mentoring offered to the health facilities had a combination of both onsite and online components. Focus of these sessions was on active problem solving by the QI teams under the guidance of national mentoring group mentors.

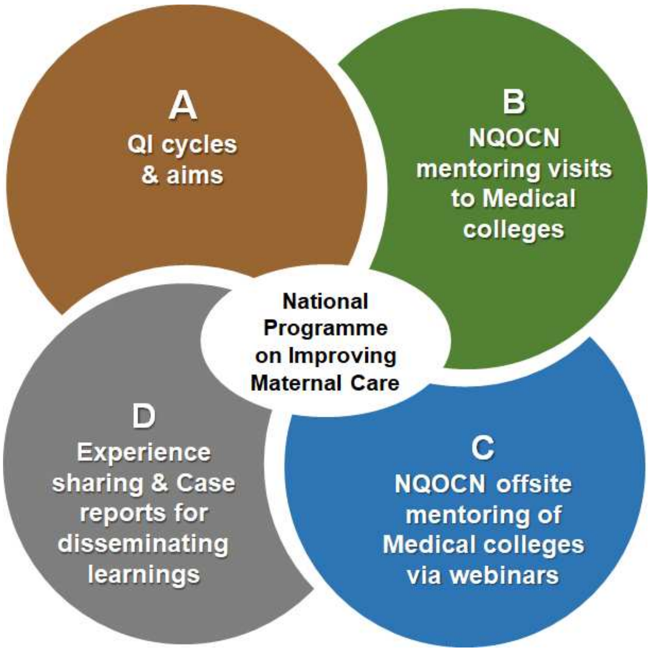

Figure g: National Mentoring Group for QI Implementation in Medical Colleges

[11] Online Community of Practice (Digital Platform) (Ongoing since Aug 2020)

The covid-19 pandemic forced the mentoring for QI to be via online mode only. This limitation, led to creation of a dedicated online platform for not only QI teams and mentors to interact but also, QI champions to share and learn from each other's experiences – known as POCQI Community of Practice (POCQI COP). Its hosted on an online platform by

the QI Network, NQOCN. It has participants from around the world (primarily from USA, UK, Qatar, Bangladesh & India). The key stakeholders involved in this COP are WHO-SEARO, Ministry of Health & Family Welfare, Govt. of India, ISQua, BMJ India, Oxford University Hospitals, NHS, University Research Company, MGIMS, Wardha, Aastrika technologies, 3M, and CAHO. The COP brings together QI champions from all facilities associated with the network, national and state health departments, development partners, QI teams from South Asia region regularly to learn from global leaders in QI and share their experiences with each other. The COP offer a unique opportunity to engage with stakeholders across all levels of the health system i.e., micro-, meso- and macro levels.

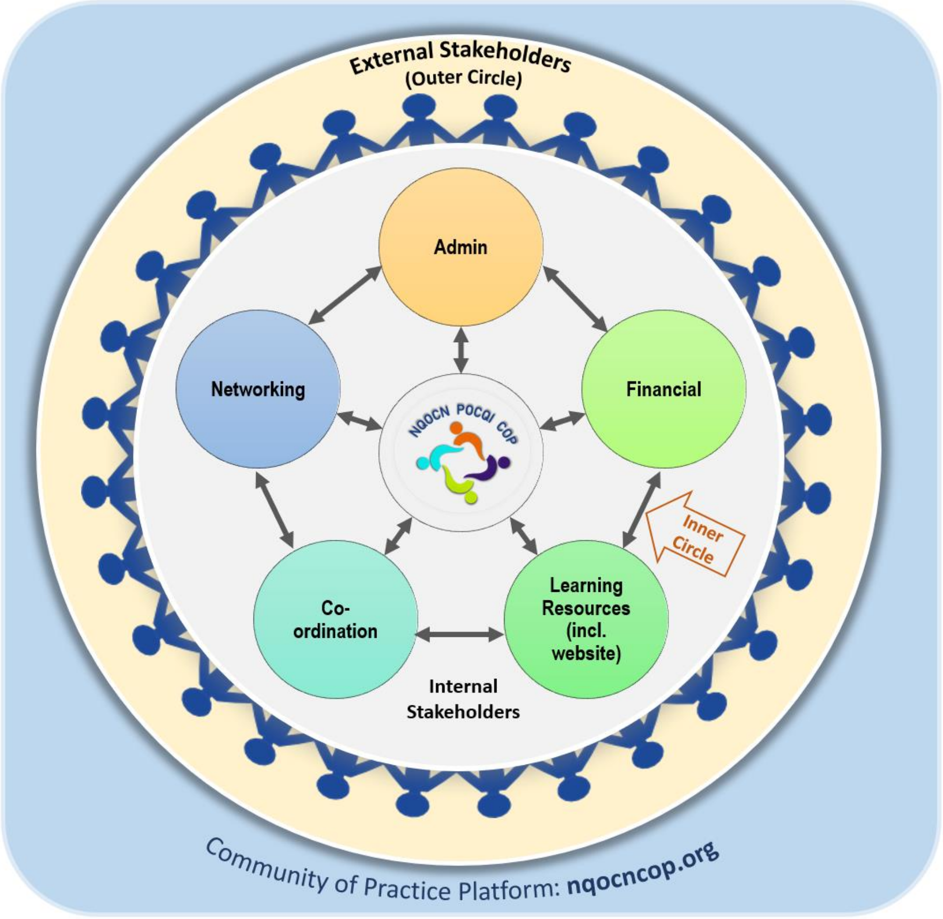

Figure h: Community of Practice on a digital platform
